# RabGTD: a comprehensive database of rabbit genome and transcriptome

**DOI:** 10.1093/database/bay075

**Published:** 2018-07-13

**Authors:** Lu Zhou, Qingyu Xiao, Jie Bi, Zhen Wang, Yixue Li

**Affiliations:** 1 Key Lab of Computational Biology, CAS-MPG Partner Institute for Computational Biology, Shanghai Institutes for Biological Sciences, Chinese Academy of Sciences, 320 Yueyang Rd., Xuhui District, Shanghai 200031, China; 2 University of Chinese Academy of Sciences, 52 Sanlihe Rd., Xicheng District, Beijing 100049, China; 3 Shanghai Center for Bioinformation Technology, Shanghai Industrial Technology Institute, 1278 Keyuan Rd., Pudong District, Shanghai 201203, China; 4 Collaborative Innovation Center for Genetics and Development, Fudan University, 2005 Songhu Rd., Yangpu District, Shanghai 200433, China

## Abstract

The rabbit is a very important species for both biomedical research and agriculture animal breeding. They are not only the most-used experimental animals for the production of antibodies, but also widely used for studying a variety of human diseases. Here we developed RabGTD, the first comprehensive rabbit database containing both genome and transcriptome data generated by next-generation sequencing. Genomic variations coming from 79 samples were identified and annotated, including 33 samples of wild rabbits and 46 samples of domestic rabbits with diverse populations. Gene expression profiles of 86 tissue samples were complied, including those from the most commonly used models for hyperlipidemia and atherosclerosis. RabGTD is a web-based and open-access resource, which also provides convenient functions and friendly interfaces of searching, browsing and downloading for users to explore the big data.

Database URL: http://www.picb.ac.cn/RabGTD/

## Introduction

The rabbit is one of the most frequently used animal models because they are mild-tempered and easy to handle, confine and breed. Besides, they are commonly used for toxicity and safety testing of substances such as drugs, chemicals and medical devices (1). A number of rabbit models have been developed to study human diseases, the most common being cardiovascular diseases (2), cancer and AIDS (3). In particular, the rabbit model contributed tremendously to the discoveries of low-density lipoprotein receptor deficiency as a cause for human familial hypercholesterolemia and statins as the most potent lipid-lowering drug (4). They have also been used as bioreactors for the production of pharmaceutical proteins such as polyclonal antibodies (5), which are commonly used in a variety of research methodologies.

The rapid development of next-generation sequencing (NGS) technology has facilitated the generation of massive rabbit genome and transcriptome datasets. A high-quality reference genome of the rabbit was published in 2012 (6). Whole-genome sequencing of a wide range of wild populations and domestic breeds was also performed to understand the genetic basis during rabbit domestication (6). Meanwhile, the targeted regions of two rabbit subspecies were sequenced to focus on their divergence by another published research (7). In our previous study, we provided the whole genome and transcriptome of the most popular experimental rabbits, especially those related to hyperlipidemia and atherosclerosis (8). In view of the wide usage of the rabbit models, it is really helpful to build an integrated database to make these data more accessible for many studies.

Here, we presented RabGTD, the first database to collect, process and display all published data of the rabbit genome and transcriptome generated by NGS. We also implemented functions of searching, overviewing, browsing and downloading of data. In total, the genome data came from 79 samples, including 33 samples of wild rabbits and 46 samples of domestic rabbits. Genomic variants (SNPs and small INDELs) and their functional annotations for a given sample could be retrieved according to the chromosomal location or gene names. The transcriptome data came from 86 tissue samples of commonly used laboratory models, of which 76 samples were from our previous work focusing on hyperlipidemia and atherosclerosis (8), and 10 samples were from another research focusing on rabbit domestication (6). Gene expression values represented by the raw and normalized read counts could be investigated. Furthermore, JBrowse (9) was implemented to explore the variants and expressions for any chromosomal locations in detail. All the data can be freely downloaded from our database.

## Database construction

### Data sources

All the DNA or RNA sequencing data reported by three previous studies (6–8) were collected from the NCBI SRA database (10). Considering different experiment design and sequencing strategies, we separated the genomic data into four batches and transcriptomic data into two batches. For the genomic data, Batches 1 and 4 adopted whole-genome sequencing, while Batches 2 and 3 were capture-based sequencing. Batches 1, 2 and 3 were comprised of individual samples and Batch 4 was pooled samples. In Batch 1, there were three popular laboratory breeds including the NZW (New Zealand white), JW (Japanese white) and WHHL (Watanabe heritable hyperlipidemic) rabbits (8). In Batch2, there were two subspecies of wild rabbits from Iberian Peninsula divided by geographical position, where one was *Oryctolagus cuniculus algirus* (*Oc. algirus*) on the northeast of the peninsula and the other was *Oryctolagus cuniculus cuniculus* (*Oc. cuniculus*) on the southwest (7). Batches 3 and 4 came from the same source (6), including six domestic breeds (Belgian Hare, Champagne, Flemish Giant, French Angora, French Lop and Rex) and one wild population [French wild (FRW)] in Batch 3, as well as seven domestic breeds (Belgian Hare, Champagne, Dutch, Flemish Giant, French Lop, Netherland Dwarf and New Zealand) and two wild populations [FRW and Iberian Peninsula wild (IW)] in Batch 4. For the transcriptomic data, breeds, tissues and experimental treatments were considered for the batch division. Batch 1 involved five tissues (aorta, heart/coronary, kidney, liver and embryo) from NZW, JW and WHHL, where NZW were treated with standard chow and cholesterol-rich diet, respectively (8). Batch 2 was sampled from 10 tissues (ovary, lung, liver, skeletal muscle, testis, heart, blood, brain, skin and kidney) of NZW without repetition (6). A detailed description about all the samples was given in Table 1.

**Table 1.** Detailed description of the data sources

Genome/ transcriptome	Batch	Sequencing strategy	Individual/pool	Domestic/wild	Breed	Tissue and treatment	Sample size	Data citation
Genome	1	Whole-genome sequencing	Individual	Domestic	JW	–	10	[8]
Genome	1	Whole-genome sequencing	Individual	Domestic	NZW	–	11	
Genome	1	Whole-genome sequencing	Individual	Domestic	WHHL	–	10	
Genome	2	Targeted capture-based sequencing	Individual	Wild	*Oc. algirus*	–	6	[7]
Genome	2	Targeted capture-based sequencing	Individual	Wild	*Oc. cuniculus*	–	6	
Genome	3	Targeted capture-based sequencing	Individual	Domestic	Belgian Hare	–	1	[6]
Genome	3	Targeted capture-based sequencing	Individual	Domestic	Champagne	–	1	
Genome	3	Targeted capture-based sequencing	Individual	Domestic	Flemish Giant	–	2	
Genome	3	Targeted capture-based sequencing	Individual	Domestic	French Angora	–	2	
Genome	3	Targeted capture-based sequencing	Individual	Domestic	French Lop	–	1	
Genome	3	Targeted capture-based sequencing	Individual	Domestic	Rex	–	1	
Genome	3	Targeted capture-based sequencing	Individual	Wild	FRW	–	7	
Genome	4	Whole-genome sequencing	Pool	Domestic	Belgian Hare	–	1	
Genome	4	Whole-genome sequencing	Pool	Domestic	Champagne	–	1	
Genome	4	Whole-genome sequencing	Pool	Domestic	Dutch	–	1	
Genome	4	Whole-genome sequencing	Pool	Domestic	Flemish Giant	–	1	
Genome	4	Whole-genome sequencing	Pool	Domestic	French Lop	–	1	
Genome	4	Whole-genome sequencing	Pool	Domestic	Netherland Dwarf	–	1	
Genome	4	Whole-genome sequencing	Pool	Domestic	New Zealand	–	1	
Genome	4	Whole-genome sequencing	Pool	Wild	FRW	–	3	
Genome	4	Whole-genome sequencing	Pool	Wild	IW	–	11	
Transcriptome	1	Whole-genome sequencing	Individual	Domestic	JW	Aorta	4	[8]
Transcriptome	1	Whole-genome sequencing	Individual	Domestic	JW	Heart	4	
Transcriptome	1	Whole-genome sequencing	Individual	Domestic	JW	Kidney	4	
Transcriptome	1	Whole-genome sequencing	Individual	Domestic	JW	Liver and embryo	4	
Transcriptome	1	Whole-genome sequencing	Individual	Domestic	NZW	Aorta	4	
Transcriptome	1	Whole-genome sequencing	Individual	Domestic	NZW	Heart/coronary	4	
Transcriptome	1	Whole-genome sequencing	Individual	Domestic	NZW	Kidney	4	
Transcriptome	1	Whole-genome sequencing	Individual	Domestic	NZW	Liver and embryo	4	
Transcriptome	1	Whole-genome sequencing	Individual	Domestic	NZW	Embryo	4	
Transcriptome	1	Whole-genome sequencing	Individual	Domestic	NZW (high cholesterol diet)	Aorta	4	
Transcriptome	1	Whole-genome sequencing	Individual	Domestic	NZW (high cholesterol diet)	Heart	4	
Transcriptome	1	Whole-genome sequencing	Individual	Domestic	NZW (high cholesterol diet)	Heart/coronary	4	
Transcriptome	1	Whole-genome sequencing	Individual	Domestic	NZW (high cholesterol diet)	Kidney	4	
Transcriptome	1	Whole-genome sequencing	Individual	Domestic	NZW (high cholesterol diet)	Liver and embryo	4	
Transcriptome	1	Whole-genome sequencing	Individual	Domestic	WHHL	Aorta	4	
Transcriptome	1	Whole-genome sequencing	Individual	Domestic	WHHL	Heart	4	
Transcriptome	1	Whole-genome sequencing	Individual	Domestic	WHHL	Kidney	4	
Transcriptome	1	Whole-genome sequencing	Individual	Domestic	WHHL	Liver and embryo	4	
Transcriptome	2	Whole-genome sequencing	Individual	Domestic	NZW	Ovary	1	[6]
Transcriptome	2	Whole-genome sequencing	Individual	Domestic	NZW	Lung	1	
Transcriptome	2	Whole-genome sequencing	Individual	Domestic	NZW	Liver	1	
Transcriptome	2	Whole-genome sequencing	Individual	Domestic	NZW	Skeletal muscle	1	
Transcriptome	2	Whole-genome sequencing	Individual	Domestic	NZW	Testis	1	
Transcriptome	2	Whole-genome sequencing	Individual	Domestic	NZW	Heart	1	
Transcriptome	2	Whole-genome sequencing	Individual	Domestic	NZW	Blood	1	
Transcriptome	2	Whole-genome sequencing	Individual	Domestic	NZW	Brain	1	
Transcriptome	2	Whole-genome sequencing	Individual	Domestic	NZW	Skin	1	
transcriptome	2	Whole-genome sequencing	Individual	Domestic	NZW	Kidney	1	

### Data processing

**Figure 1.**
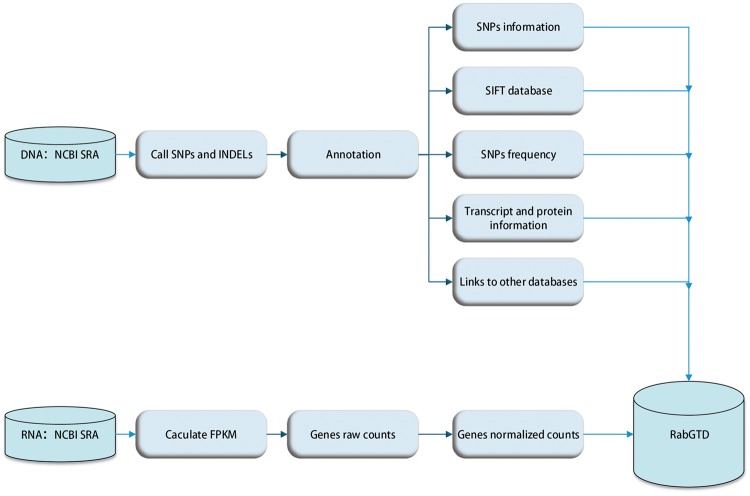
The flow diagram of data preprocessing.

The raw sequencing data were processed with standard pipeline uniformly (Figure 1). For genome sequencing, raw reads passing quality check were mapped to the rabbit reference genome (oryCun2) using BWA-MEM [v0.7.4] (11), SNPs, small INDELs and genotypes were called by SAMtools [v0.1.9] with default parameters (12), and the genotype of each sample was assigned by VCFtools [v0.1.11] (13). Functional effects of the variants were annotated by ANNOVAR [v2013-06-21] (14), using the Ensembl [v76] (15) genes as the reference. For all variants, genic locations and corresponding gene information were given. For those located within the exonic regions, the amino acid mutations were also inferred. In addition, conservation scores of the non-synonymous SNPs were retrieved from the SIFT (16) database, where an amino acid substitution was predicted to cause protein function damage with the SIFT score < 0.05. We also calculated the allele frequency of each variant in the population of wild and domestic rabbits, respectively.

For RNA-Seq data, raw reads were filtered by NGS QC Toolkit [v2.3.2] (17) and mapped to the reference genome (OryCun2) by TopHat2 [v2.0.8] (18). The transcripts were assembled and merged by Cufflinks [v2.0.2] (19), guided by the Ensembl (15) annotations. The raw read counts of genes were counted by HTSeq (v0.6.0) (20), which were then normalized by DESeq [v1.16.0] (21) to facilitate comparison among samples (Figure 1).

### Database implementation

RabGTD can be accessed through web browsers (http://www.picb.ac.cn/RabGTD/). A web browser version higher than Chrome 15, Internet Explorer 10, Firefox 8, Opera 12 or Safari 8 will work fine for the RabGTD. The web application was developed with jQuery [version 2.2.2] as a web-based front end, PHP [version PHP 7.0.7] as a back end, and Mysql [version 14.14] as a data management system. The DNA variants and RNA expression values of all the samples are available to search. In addition, the JBrowse [version 1.12.1] (9) plugin is used as a genome browser with a fully dynamic ajax interface, which provides a visualization interface of more detailed information for a given genomic region. All the data explained above can also be freely downloaded.

## Database features and utilizations

### Search variants

**Figure 2.**
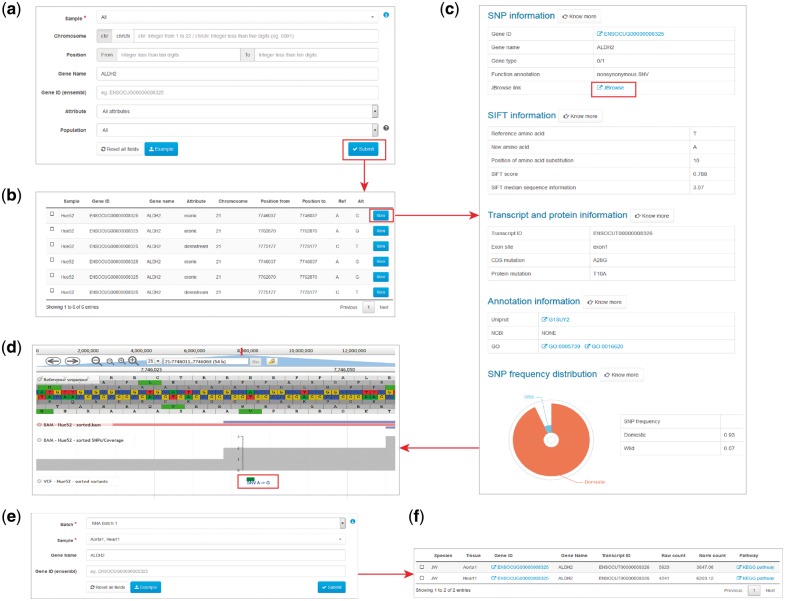
Examples of variant and expression search. (**a**) Entry of variant search. (**b**) Results of variant search. (**c**) Extended results of variant search. (**d**) Display of the variant with JBrowse. (**e**) Entry of expression search. (**f**) Result of expression search.

The ‘Search variants’ feature allows to search genomic variants for a selected sample (Figure 2a). The sample option is required to be chosen, which are summarized in the data information page of our website. There are two alternative ways to search variants. One is to specify the genomic locations of the variants, including the chromosome number and the interval of physical distance in base pairs. If the variants are located on scaffold sequences, the ‘chrUN’ tag should be chosen. The other is to search the variants through gene name or Ensembl gene ID. In addition, user can select the genic attribute of the variants or population to filter the searching result. To speed up the searching process, only variants with attributes closely related to gene functions such as exonic, splicing, UTR5, UTR3, upstream, downstream could be searchable.

The search result of variants is displayed as a table, containing the basic information of sample, gene name, attributes, genomic position, reference and alterative alleles (Figure 2b). More information can be obtained for each variant in an extended panel, including the information of SNP and genotype, SIFT score, alteration in transcript and protein, gene function and variant frequency in domestic and wild rabbits (Figure 2d). External links to the Uniprot, NCBI and GO database for the investigated gene are also provided.

### Search expressions

The ‘Search expressions’ feature allows to search gene expression values for selected samples. Batch and sample, along with tissue should be chosen at first, which could all be found in the data information page of our website. Search through gene name or Ensembl gene ID are both supported for this feature (Figure 2e). The search result succinctly provides information of gene name, transcript ID, raw and normalized read counts, pathway (22) (Figure 2f).

### Genome browser

The JBrowse plug-in is accessible from RabGTD to visualize the genome and transcriptome data in more details. We have imported the reference genome sequence, reference sequence annotation (.gtf file), genomic variants of each sample (.vcf files), reads mapped to the reference (.bam files) as well as sequencing coverage to the genome browser. All genomic variants including intergenic and intronic ones could be browsed here. Each of files could be selected to display as a separate track (Figure 2c). Users can locate the genomic regions they are interested in to visualize the sequencing data. More features of JBrowse could be found at https://jbrowse.org.

### Data downloads

All the genome and transcriptome data for every sample in the database are available for download, including the bam and vcf files. The data information page provides external links to download raw sequencing data from NCBI SRA database.

### Data analysis

The overall statistics of the genomic variants and gene expressions are summarized in the chart page of our website. Here, we integrated the genomic variants from different batches for a comprehensive investigation of the rabbit domestication.

**Figure 3.**
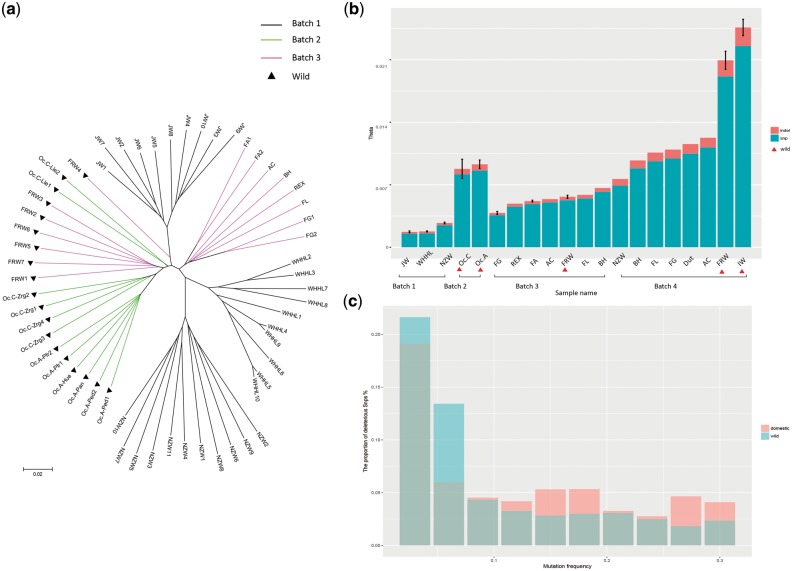
Analysis of rabbit domestication by data integration. (**a**) The Watterson’s theta based on SNPs and INDELs of all samples. The wild samples are labeled by red triangles. For breeds with more than one samples, we calculated the standard deviation. (**b**) The phylogenetic tree of individual samples from Batches 1–3. Batches are indicated by different colors and the wild samples are labeled by black triangles. (**c**) The proportion of deleterious SNPs with different mutation allele frequency for domestic and wild samples, respectively.

First, we constructed a neigbor-joining tree for the wild and domestic individuals from DNA Batches 1–3 based on their pairwise differences (Figure 3a). Generally, we found that the wild and domestic breeds could be separated into two distinct clades across batches. All of the two wild subspecies from Batch 2 (*Oc. cuniculus* and *Oc. algirus*) and most of the wild ones from Batch 3 (FRW) were grouped into the same clade. The three domestic breeds from Batch 1 (JW, WHHL and NZW) were grouped with a variety of domestic breeds from Batch 3, suggesting a single origin of all domestic rabbits. This result was consistent with our knowledge of rabbit domestication (23) and illustrated that the data across batches could be aggregated for a more comprehensive analysis.

Next, we calculated the genetic diversity (measured by Watterson’s theta) of all the samples (Figure 3b). We made a comparison between the wild and domestic rabbits among the individual samples (DNA Batches 1–3) and pooled samples (Batch 4), respectively. As the original report (6), in Batch 4 we observed a first reduction in the genetic diversity when the wild rabbits from the Iberian Peninsula (IW) migrated to southern France (FRW), and then a second reduction during domestication which created other European domestic breeds. Among the individual samples based on whole-genome sequencing, we confirmed that the wild samples from Iberian Peninsula (Batch 2) had the highest genetic diversity and the three laboratory breeds in Batch 1 had the lowest genetic diversity. But in Batch 3, the genetic diversity of FRW was not always higher than that of the domestic breeds, which might be caused by the limited variants from capture-based sequencing. Altogether, an explicit comparison of our result showed high consistency with previous published works (Supplementary Table S1).

Finally, we compared the frequencies of deleterious mutations between the wild and domestic individual samples based on the SIFT score. In total, there were 12.72% deleterious SNPs in the wild rabbits, while there were 14.32% deleterious SNPs in the domestic rabbits. We further considered the deleterious mutations with different mutation allele frequency (MAF) (Figure 3c). Although the deleterious mutations were both enriched in low MAF, the proportion of deleterious mutations with high MAF (>0.1) was still larger in the domestic rabbits than that in the wild rabbits. The easier accumulation of deleterious mutations in the domestic rabbits could be triggered by two main factors: a relaxation of selective constraints due to a population bottleneck and altered breeding patterns accompanying domestication (24–26), as well as an effect of positive selection at linked sites, which reduced the probability that slightly deleterious mutations would be purged from the population (27, 28). This result was reported in rabbits for the first time, which suggested that novel findings could be derived from our database.

## Discussion

To our knowledge, RabGTD is the first comprehensive rabbit database comprising both genome and transcriptome data. It collected all available data from rabbit sequencing projects up to now and provided convenient functions and friendly interfaces for users to explore the big data. By integrating the genomic data, our analysis demonstrated that our database could be used to reveal the domestication process of rabbits. Besides, RabGTD will also facilitate the use of rabbits in many biomedical studies for human diseases and transgenic models. Moreover, our database is also extendable to accommodate the rapid growth of sequencing data for rabbits.

## Supplementary data

Supplementary data are available at *Database* Online.

## Funding

National Key R&D Program of China (2016YFC0901704 and 2017YFA0505500); and the Youth Innovation Promotion Association CAS (2017325).

*Conflict of interest*. None declared.

## Supplementary Material

Supplementary Data
