# Supplementary material for: RabGTD: a comprehensive database of rabbit genome and transcriptome
Source: Database (Oxford). 2018 Jul 13;2018:bay075. doi: 10.1093/database/bay075 (PMC6047408; doi:10.1093/database/bay075)
Supplement: Supplementary Data [file bay075_supp.docx]

**Supplementary**

**Table S1** The comparison of the genetic diversity estimated by our research and previous reported study.

| **Batch** | **Abbreviation** | **Full name** | **Domestic/Wild** | | **Pi*** | **Pi reported in previous study(**[**1**](#_ENREF_1)**)** |
| --- | --- | --- | --- | --- | --- | --- |
| 1 | JW | Japanese White | Domestic | 0.0043 | |  |
| 1 | WHHL | Watanabe heritable hyperlipidemic | Domestic | 0.0046 | |  |
| 1 | NZW | New Zealand White | Domestic | 0.0049 | |  |
| 2 | Oc.C | Oryctolagus cuniculus cuniculus | Wild | 0.0027 | |  |
| 2 | Oc.A | Oryctolagus cuniculus algirus | Wild | 0.0027 | |  |
| 3 | FG | Flemish giant | Domestic | 0.0026 | |  |
| 3 | REX | REX | Domestic | 0.0025 | |  |
| 3 | FA | French Angora | Domestic | 0.0026 | |  |
| 3 | AC | Champagne d'Argent | Domestic | 0.0026 | |  |
| 3 | FRW | French Wild | Wild | 0.0026 | |  |
| 3 | FL | French lop | Domestic | 0.0026 | |  |
| 3 | BH | Belgian Hare | Domestic | 0.0026 | |  |
| 4 | NZW | New Zealand White | Domestic | 0.0056 | | ~0.0026 |
| 4 | BH | Belgian Hare | Domestic | 0.0058 | | ~0.0031 |
| 4 | FL | French lop | Domestic | 0.0061 | | ~0.0038 |
| 4 | FG | Flemish giant | Domestic | 0.006 | | ~0.0035 |
| 4 | Dut | Dutch | Domestic | 0.0059 | | ~0.0038 |
| 4 | AC | Champagne d'Argent | Domestic | 0.0061 | | ~0.004 |
| 4 | FRW | French Wild | Wild | 0.0072 | | ~0.0060 |
| 4 | IW | Iberian Peninsula Wild | Wild | 0.0084 | | ~0.0080 |
| **Pi***: A commonly used measure of nucleotide diversity ([2](#_ENREF_2)) . | | | | | | |

**References**

1. Carneiro, M., Rubin, C.J., Di Palma, F.*, et al.* (2014) Rabbit genome analysis reveals a polygenic basis for phenotypic change during domestication. *Science*, **345**, 1074-1079.

2. Nei, M., Li, W.H. (1979) Mathematical model for studying genetic variation in terms of restriction endonucleases. *Proceedings of the National Academy of Sciences of the United States of America*, **76**, 5269-5273.
